# Clinical Epigenomic Explanation of the Epidemiology of Cannabinoid Genotoxicity Manifesting as Transgenerational Teratogenesis, Cancerogenesis and Aging Acceleration

**DOI:** 10.3390/ijerph20043360

**Published:** 2023-02-14

**Authors:** Albert Stuart Reece, Gary Kenneth Hulse

**Affiliations:** 1 Division of Psychiatry, University of Western Australia, Crawley, WA 6009, Australia; 2 School of Medical and Health Sciences, Edith Cowan University, Joondalup, WA 6027, Australia

**Keywords:** tobacco, alcohol, cannabis, cannabinoid, cancer, cancerogenesis, mutagenesis, oncogenesis, genotoxicity, epigenotoxicity, transgenerational inheritance

## Abstract

As global interest in the therapeutic potential of cannabis and its’ derivatives for the management of selected diseases increases, it is increasingly imperative that the toxic profile of cannabinoids be thoroughly understood in order to correctly assess the balance between the therapeutic risks and benefits. Modern studies across a number of jurisdictions, including Canada, Australia, the US and Europe have confirmed that some of the most worrying and severe historical reports of both congenital anomalies and cancer induction following cannabis exposure actually underestimate the multisystem thousand megabase-scale transgenerational genetic damage. These findings from teratogenic and carcinogenic literature are supported by recent data showing the accelerated patterns of chronic disease and the advanced DNA methylation epigenomic clock age in cannabis exposed patients. Together, the increased multisystem carcinogenesis, teratogenesis and accelerated aging point strongly to cannabinoid-related genotoxicity being much more clinically significant than it is widely supposed and, thus, of very considerable public health and multigenerational impact. Recently reported longitudinal epigenome-wide association studies elegantly explain many of these observed effects with considerable methodological sophistication, including multiple pathways for the inhibition of the normal chromosomal segregation and DNA repair, the inhibition of the basic epigenetic machinery for DNA methylation and the demethylation and telomerase acceleration of the epigenomic promoter hypermethylation characterizing aging. For cancer, 810 hits were also noted. The types of malignancy which were observed have all been documented epidemiologically. Detailed epigenomic explications of the brain, heart, face, uronephrological, gastrointestinal and limb development were provided, which amply explained the observed teratological patterns, including the inhibition of the key morphogenic gradients. Hence, these major epigenomic insights constituted a powerful new series of arguments which advanced both our understanding of the downstream sequalae of multisystem multigenerational cannabinoid genotoxicity and also, since mechanisms are key to the causal argument, inveighed strongly in favor of the causal nature of the relationship. In this introductory conceptual overview, we present the various aspects of this novel synthetic paradigmatic framework. Such concepts suggest and, indeed, indicate numerous fields for further investigation and basic science research to advance the exploration of many important issues in biology, clinical medicine and population health. Given this, it is imperative we correctly appraise the risk–benefit ratio for each potential cannabis application, considering the potency, severity of disease, stage of human development and duration of use.

## 1. Introduction

Cannabinoid genotoxicity is not controversial and is widely acknowledged by both government drug regulators and the cannabis industry. The official prescribing information for both Sativex (Δ9-tetrahydrocannabinol (THC)—cannabidiol) and Epidiolex (cannabidiol) registered with the US Food and Drug Administration (FDA), the European Medicines Agency (EMA) and the Medicines and Health Care Products Regulatory Agency (MHCPRA) of the UK [1,2] carry warnings against the use of these products in pregnancy and lactation. Further, some products released directly by the cannabis industry, and thus not formally regulated, contain similar reproductive warnings.

However, while cannabinoid genotoxicity has been well established experimentally in a variety of different in vitro systems [3,4,5,6,7,8,9,10,11,12,13,14,15,16,17,18,19], this information is strikingly absent from most public health discussions on the place of cannabinoids and the management of morbidity. The public health implications of the documented cannabinoid genotoxicity have more recently begun to be appreciated with large, newly published epidemiological investigations of cannabinoid-related cancerogenesis, teratogenesis, heritable teratogenic cancerogenesis and the acceleration of aging.

With rising interest in the pharmaceutical use of cannabis for the management of selected diseases and a strong commercial and popular interest in the therapeutic potential of cannabis derivatives, it becomes increasingly important that the toxic profile of cannabinoids is thoroughly understood in order to parse the classical balance between the therapeutic risks and benefits correctly and astutely. Inherently, this risk–benefit ratio must be correctly appraised for each potential cannabis application, considering the potency, severity of disease, stage of human development and duration of use. 

The correct and astute appraisal of cannabinoid toxicity becomes even more important in the case of cannabinoids where, at the time of writing, reports from both the Institute of Medicine and the National Academy of Sciences described the objective evidentiary basis underpinning cannabis prescription as often “weak” to “moderate at best”, and most of its hypothetical indications as being of anecdotal level only, and so of weak evidentiary power [20,21].

Contrariwise, strong epidemiological evidence from many jurisdictions showed the likely involvement of cannabinoids in many cancers, dozens of serious birth defects and cellular and organismal aging [22,23,24,25,26,27,28,29,30,31,32,33,34,35,36,37,38,39,40,41,42,43,44,45,46,47,48,49,50,51,52,53,54]. Moreover, there is a remarkably close uniformity between the findings from different jurisdictions [38,39], which not only confirms the findings of single studies but also, according to the established Hill criteria of causality, lends formal credence to the likely causal relationship between epidemiologically assessed cannabis exposure and the observed morbidity.

While it is acknowledged that some historical literature assessing the cannabis morbidity associations produced data showing no effect, the field is plagued with numerous methodological difficulties, including the conduct of many widely quoted studies in earlier eras when cannabis was of a much lower THC potency and the increase in the community exposure since that time, in terms of not only the prevalence of cannabis consumption but the intensity of cannabis consumption. In some widely quoted studies [55], the systematic deletion of individuals who had experienced a high dose exposure was practiced. Clearly, it is in patients with this higher exposure level where more significant effects might reasonably be expected. The issue of small sample sizes is also a major and common shortcoming of many studies. It should also be noted that relatively sudden increases in cannabis use prevalence, THC potency and cannabis daily use intensity all occurring at once will launch the community relatively abruptly into the higher exposure levels where, due to the exponential dose-response relationship for many genotoxic [3,5,6,7,8,10,11,14,16,18,19] and mitochondrial metabolic [4,9,12,13,15,17] consequences, the negative outcomes become both more common and more severe.

Importantly, in attaining a cannabis dose-response threshold, there has been an increase in the cannabis exposure for some communities, arising not only from an increased prevalence in cannabis consumption but from an increased potency and frequency of use [56,57], where the well-documented exponential genotoxic dose-response effect curves shown in many laboratory [3,5,6,7,8,10,11,14,16,18,19] and preclinical animal model assays [58,59,60] take effect. Since the cannabis use prevalence, intensity of daily use and THC potency are all rising *simultaneously,* these features can be expected to operate synergistically as a relatively abrupt switch where severe adverse genotoxic and neurotoxic outcomes relatively suddenly become commonplace due to the underlying exponential dose-response effects [56,57].

It is well established that the trend of cannabinoid exposure in both the US and Europe has been upwards and positive (increasing) in recent decades. This rising trend carries two major implications. Firstly, in the US, it has been shown that the prevalence of cannabis use has increased, the rate of daily cannabis use has more than doubled [61,62] and the THC content of cannabis has increased from 3.4% in 1993 to 17.1% in 2017 [63,64,65,66]. Similar changes are reported in Europe [56,57]. Moreover, it has also been shown that the combination of these three metrics (of the prevalence, intensity and THC concentration) is the most powerful predictor of the teratogenic genotoxic outcomes [40]. Were these three changes occurring in isolation, they would be cause for concern in themselves. However, their occurrence in many places *simultaneously* implies a major paradigmatic shift in the community exposure from a relatively low level to a much higher region where genotoxic outcomes become more common.

Secondly, it would be expected that the rising trend in cannabinoid exposure shows a simple bivariate statistical correlation with any rising trend in the population with ill health. Accordingly, recent research has established a robust relationship with multisystem tumorigenesis and teratogenesis, which is robust to multivariable adjustment, is verified in combined formal spatiotemporal analyses and satisfies the modern quantitative criteria of causality with very similar results reflected in both the US and European datasets [22,23,24,25,26,27,28,29,30,31,36,37,40,41,44,45,46,67].

It must be underscored that the triple convergence of cannabinoid carcinogenesis, cannabinoid teratogenesis and the cannabinoid acceleration of aging together forms strong and theoretically robust evidence for a clinically and highly significant genotoxicity [68] severe enough to impact numerous metrics of the population health adversely. 

Furthermore, both in vitro and clinical studies implicate many different cannabinoid moieties, suggesting that genotoxicity is a class effect shared by many cannabinoids [69,70]—a feature now well confirmed by many epidemiological studies. This includes such allegedly benign cannabinoid species as Δ9THC, Δ8THC and cannabidiol, among several others [22,23,24,28,71,72].

For the reader who is unfamiliar with this epidemiological literature, it should be pointed out that most of the modern epidemiological studies referred to are not just observational ecological studies of convenience which happen to show a particular association. Many of the best studies used a formal space–time analysis and the quantitative tools of causal inference to introduce a pseudo-randomized quasi-experimental paradigm from which it is entirely appropriate to invoke causal associations [28,36,37,40,45,51,71,73,74,75,76,77,78,79,80,81,82].

It is the purpose of the present paper to explore the manner in which the diverse recent laboratory and clinical results, which are the outcomes of the three primary expressions of cannabinoid genotoxicity in cancerogenesis, teratogenesis and aging, are explained using cannabinoid epigenotoxicity and the mechanistic insights which follow an improved understanding and appreciation of the magnitude and breadth of the scope of the epigenotoxic profile of cannabinoids. Since this is only beginning to be explored, the present review is necessarily limited to an introductory oversight, but it does lead to the formulation of a broad ranging experimental investigation into the ways in which these insights can be developed and explored further with far reaching implications across the spectrum of clinical medicine and the basic biological sciences.

## 2. Cannabinoid Genotoxic Phenomenology

### 2.1. Cannabinoid Genotoxic Carcinogenesis

There is impressive overlap between the US and European data for cannabis exposure and tumors. A recent review of 28 US cancer tumors that were significantly associated with Δ9THC included acute myeloid leukemia, breast, oropharynx, thyroid, liver, pancreas, chronic myeloid leukemia, testis and kidney [22]. The cancers which were significantly associated with cannabidiol were prostate, bladder, ovary, all cancers, colorectum, Hodgkin’s, brain, non-Hodgkin’s lymphoma, esophagus, breast and stomach [22,23,24]. Eight cancers significantly associated with Δ8THC on bivariate testing included corpus uteri, liver, gastric cardia, breast and post-menopausal breast, anorectum, pancreas and thyroid [72]. An additional 18 tumors demonstrated positive marginal effects after the multivariable adjustment, including stomach, Hodgkin’s and non-Hodgkin’s lymphomas, ovary, cervix uteri, gall bladder, oropharynx, bladder, lung, esophagus, colorectal cancer and all cancers (excluding non-melanoma skin cancer) [72].

Similarly, in a review of 40 European cancers, 27 tumors were related to various metrics of cannabis exposure, including daily use. The tumor overlap included all cancers (excluding non-melanoma skin cancer), oropharynx, the four major leukemias and Hodgkin’s and non-Hodgkin’s lymphoma, liver, pancreas, brain medulloblastoma, anus, kidney, thyroid, testis (seminoma and non-seminoma), ovary and ovarian germ cell tumors. They also identified hepatocellular, skin melanoma, mesothelioma, Kaposi sarcoma, penis, prostate, vulva and vaginal cancers [83].

Hence, there is impressive overlap between the US and European data for those tumors which are listed as common.

It is mechanistically noteworthy that, for several of the tumors aforementioned, chromosomal translocation is an important and well-established pathway in their oncogenesis. The presumed mode of action here is to constitutively activate the proto-oncogenes or suppress the tumor suppressor genes. These comments apply particularly to acute myeloid and lymphoid leukemias (AML and ALL) and to testicular cancer [25,84,85,86,87]. Indeed, if one adds up all the chromosomes commonly implicated in ALL and testicular cancer, they result in 1254 megabases and 645 megabases, which represent 41.8% and 21.5% of the 3000 megabases of the human genome, respectively.

### 2.2. Cannabinoid Genotoxic Teratogenesis

When 62 congenital anomalies were tracked longitudinally across the US, 45 were shown to be related to the metrics of cannabis exposure, particularly those from the cardiovascular, chromosomal, gastrointestinal, limb, urinary, body wall and face [28].

When a series of 95 European congenital anomalies were studied, 89 were shown to be relatable to the various metrics of cannabis exposure and were related particularly to the anomalies affecting the cardiovascular, gastrointestinal, uronephrological and central nervous systems, as well as the chromosomal, limb, body wall, face and general (unallocated) [40].

Again, a significant overlap was shown where the anomalies listed were in common [36].

It is of interest that if one adds the lengths of the chromosomes directly impacted by these chromosomal anomalies together, it results in 388 of the 3000 megabases of the human genome, or 12.9%.

### 2.3. Cannabinoid Genotoxic Aging Acceleration

The data from the twelve separate empirical streams both independently and more strongly collectively provide a convincing empirical case for cannabis-induced accelerated aging. These include hepatotoxic [88], immunological [89,90,91,92,93,94,95,96,97,98,99], genotoxic [3,5,6,7,8,10,11,14,16,18,19], epigenotoxic [42,100,101,102,103,104,105,106,107,108], disruption of chromosomal physiology [109,110,111,112,113,114,115], endocrine [116,117,118,119,120], congenital anomalies [28,36,40,45,46,51] and cancers [22,23,24,26,30,34,41,51], including inheritable tumorigenesis [25,26], telomerase inhibition [42,121], mitochondriopathic [122,123,124,125,126], cardiovascular [33] and elevated mortality [127,128,129,130,131,132,133,134,135,136,137,138]. Cannabis dependence not only recapitulates many of the key features of aging, but is characterized by both age-defining and age-generating illnesses, including hepatoinflammatory disorders, immunomodulation, many psychiatric syndromes with a neuroinflammatory basis [139,140,141,142,143,144,145,146,147,148,149,150,151,152,153,154,155,156,157,158,159,160,161], genotoxicity [3,5,6,7,8,10,11,14,16,18,19] and epigenotoxicity [162,163,164,165,166].

A recent detailed report from a large electronic health record database in Hawaii showed that cannabis users were subject to elevated rates of an impressive array of acute illnesses, including myocardial infarction, stroke, acute bronchitis, cyclic vomiting, injuries, poisonings, car wrecks, falls and several chronic diseases, including coronary artery disease, hypertension, chronic obstructive pulmonary disease, chronic pain, behavioral health disorders, addictions and poverty [53]. Overall, this pattern of chronic ill health includes many age-defining illnesses and provides solid clinical evidence of accelerated aging [163,164,165,166].

This important study was followed by another pivotal study, which demonstrated a dramatic 30% increase in cellular aging at a median chronological age of 30 years using a late-generation epigenomic clock based on DNA methylation, demonstrating the acceleration of the aging process from cannabinoid exposure in somatic (non-germ cell) tissues utilizing ‘state-of-the-art’ technologies [54].

However, arguably of greater concern, is that fact that the nuclear blebs and bridges and chromosomal breaks and translocations well-described for sperm and oocytes in the experimental cannabis literature [114,167] are signs of advanced cellular aging [162]. This leads to the conclusion that the fertilized zygote must also be prematurely aged from the time of conception since both component parts are also aged. Given that the explosion of evidence from epigenomics has now placed the Barker hypothesis of the prenatal origins of adult disease [168,169,170,171] on a firm evidentiary basis, such a finding of cannabis-induced advanced aging carries far reaching and grave public health and transgenerational implications. 

## 3. Mechanisms of Cannabinoid Genotoxicity

Central to assigning a potentially causal relationship between the exposure and observed epidemiological effects was the presence of the plausible biological mechanisms of action [172]. The past published research is awash with a flotilla of biological mechanisms by which the cannabinoids effects in the reproductive tracts [173,174], including at the level of both the male and female gametes [173,174,175], chromosomal breaks and translocations [11,109,110,115], the nucleotide bases of DNA [11], single and double stranded DNA breaks [11], the mitochondrial metabolic machinery which forms the (small molecule co-substrates, energetic and intracellular intraorganellar signaling) basis of epigenetic regulation [122,123,124,125,126,176,177,178,179,180] and the epigenomic machinery itself [42], have all been implicated in prior studies.

While these many different modalities were explored in a number of previous publications and reviews [22,23,24,25,26,27,28,29,30,31,32,33,34,35,36,42,43,49,101,102], the extraordinary detail, predictive power and keen mechanistic insights provided by recent fine-level studies of the epigenetic changes to human sperm DNA methylation following cannabis exposure and withdrawal [42] provided a truly extraordinary insight not only into the various tumors, congenital anomalies and aging-related changes reported following cannabis exposure but also their impressive breadth, diversity and variety. Importantly, these methylation changes to the human sperm epigenome showed an intricate and intimate concordance with the morbidity identified in the large-scale community cannabis exposure epidemiological studies. Paradoxically, this important feature was not readily cited or understood. For these reasons, it was of considerable importance to explore these epigenomic findings in light of the most modern and penetrating epidemiological studies.

### 3.1. Fundamental Primacy of the Epigenomic Effects

#### 3.1.1. Layers of Epigenomic Regulation

Many layers of epigenomic regulation are described and the list appears to be rapidly increasing. While they may be listed individually, they are not independent and are coordinated across the various layers. The key parameters include DNA methylation, the histone post-translational modifications, various short and long non-protein coding RNAs, over 100 post-transcriptional modifications to the RNAs, including m6-adenosine RNA methylation (also referred to as epitranscriptomics), the position with respect to the nuclear lamina (which is suppressive of the gene transcription), the chromatin state (euchromatin or suppressive heterochromatin), the presence within the transcriptional factories of the topologically defined domains (and their controlling boundary elements) and the presence of the tethering elements (especially important during the development) [181].

#### 3.1.2. Epigenomic Functions

It is now well understood that the cell lineage specification (that is, whether a cell develops as a muscle cell or a neuron, etc.) is controlled epigenomically. This issue was first formalized in the epigenetic valley hypothesis of Conrad Waddington [182]. It is also well established that the state of the cell differentiation from a pluripotent embryonic cell to a mature fully differentiated cell is also controlled epigenomically.

For a long time, the mechanisms of aging were not understood and many competing and often complimentary and overlapping theories were advanced [162]. However recent studies have confirmed that, while there are many different pathways to induce age-related damage, the major controller of cellular age is actually the epigenomic state of the cell on which other pathways likely converge [183,184,185]. Thus, robust evidence of the reversal of epigenomic clock aging, biological age and the youthful/neonatal functional capacity has now been convincingly demonstrated in many systems, including optic nerve crush injury, congenital glaucoma and ocular aging, progeroid mouse models, cardiac and skeletal muscle and fibroblasts [184,186,187,188]. This view is concordant with the well-established control of the state of the cell differentiation by the epigenomic machinery.

This implies that the epigenomic state is central and pivotal to the control of cancer, cell development and aging, which are the three principal themes of the present discussion.

### 3.2. Epigenomic Impacts of Cannabis Exposure and Withdrawal

A recent detailed epigenome-wide association study (EWAS) by Schrott and colleges investigating the DNA methylation changes of human and mouse sperm both in cannabis dependence and withdrawal provides a 359-page Supplementary Appendix listing the detailed methylation changes [42]. These researchers looked at the differential DNA methylation of the cannabis-dependent humans and mice compared to the cannabis free controls and again after an 11-week period of washout following a documented period of abstinence and detoxification from the cannabis. This longitudinal design is a very powerful way to design an epigenomic study. Close study of this dataset revealed the following remarkable findings.

#### 3.2.1. Disruption of the Epigenetic Machinery

There was widespread disruption between the main readers, writers and erasers of the epigenetic code. Hence, there were five hits for the DNA methyltransferases which added the methylation mark to the CpG islands and one hit for TET1 (ten-eleven translocase) which began the process of removing it. There was one hit for telomerase which controlled the end length of the chromosomes, and thus protected them against aging, three hits for polycomb repressors, five hits for the chromatin remodelers (SMARCA’s, SWI/SNF-related, matrix-associated, actin-dependent regulator of chromatin, subfamily A) and three hits for the UHRF (Ubiquitin-like with PHD and ring finger domains) family which controlled both DNA methylation and histone methylation and integrated the signaling in the two classes of pathways.

There were 161 hits for the histone methyltransferases with methylate histones and 199 hits for the histone demethylases that remove this mark. There were eleven hits for both the histone acetyltransferases, which acetylate histone tailed and thereby made the genome accessible to the transcription machinery, and eleven hits for the deacetylases which removed this mark.

#### 3.2.2. Stem Cell Renewal Factors

Considering the key stem cell induction factors identified by Yamanaka, Oct3/4, Sox2, Klf4 and Myc [186], all four were positively identified in this EWAS screen. When the EWAS screen was widened somewhat to include the other stem cell factors identified by the Yamanaka group and others [188], again many were positively identified, including Ras, catenins, Kit and the Lin28 microRNA.

#### 3.2.3. Chromosomal Disorders

As noted above, chromosomal disorders hold a prominent place in the patterns of cannabinoid-related carcinogenic and teratogenic disorders. Indeed, when the length of all the chromosomes involved was summed (omitting duplications), it was found that an impressive 1765 megabases of the 3000 megabases of the human genome were directly implicated in the cannabinoid-related genotoxic disorders, which is 58.8% of the human genome. For this reason, the epigenomic findings of the Schrott EWAS dataset were of immense importance.

Additionally, the rays of the mitotic spindle were composed of microtubules of polymerized tubulin. There were 106 hits in the Schrott EWAS database for tubulins. Importantly, tubulin undergoes numerous post-translational modifications which are thought to govern its intracellular trafficking and organellar addressing [189,190,191]. The cannabis withdrawal disrupted the alpha tubulin acetyl transferase which was tasked with acetylating tubulin and thereby made the microtubules flexible and increased its tensile and torsional strength. This was important as the microtubules are normally bent during the spindle formation and tensioning. Failure of completing this action leads to microtubular breaks and fractures and, thus, the chromosomal derailment during the anaphase.

The centromeres are the critical central portions of chromosomes where binding occurs to the mitotic spindle. Centrosomal protein A (CENPA) was a modified version of histone 3 (H3) and CENPA replaced H3 in the centromere, which marked the location of the centromere. Upon this CENPA basis, a complicated scaffold of 17 proteins assembled which then was bound to the microtubules of the spindle via other kinetochore scaffolding proteins [192,193,194,195,196]. Fifteen different CENPs were identified in this EWAS screen, including 86 hits for CENPN, which was the second protein to bind to the centromere complex.

The proteins which cohere the ends of the human oocyte meiotic spindle so that two (and only two) spindle poles are formed, guiding the formation of two (and only two) daughter cells, are called centrosomal organizers. There were three hits for these proteins, including the nuclear mitotic apparatus protein (NUMA).

The motor proteins which actually move the chromosomes along the microtubules towards minus end of the microtubules and the spindle poles after the anaphase checkpoint is released are called dynein motors which are controlled by a binding partner known as dynactin. There were seven EWAS hits for dynein–dynactin. Interestingly the intracellular kinesin motor moved protein and other cargo in the opposite direction towards the positive end of the microtubule and 218 hits were recorded for kinesin motors.

Sumoylation was shown to be a key post-translational modification of the key proteins, which organizes the chromosomes and are known as the remodelers of the structure of the chromatin (RSC) complexes in yeast [197]. Sumoylation involves the addition of small ubiquitin-like molecules, often in chains, to the key signaling residues of the proteins. Sumoylation of the RSC forms the founder post-translational modification upon which a string of subsequent post-translational modifications may be established [198]. These are believed to control the RSC complex activity. This RSC was shown to be centrally involved in the key chromosomal functions, such as the DNA break repair, chromosomal segregation and chromosomal duplication [198]. Δ9THC inhibited this sumoylation process directly [100], disrupting the downstream signaling through the epigenetic histone code to H3 mono-, di- and tri-methylation, H3/H4 acetylation and H2B lysine 123 ubiquitylation [198]. 

It was also noted that the EWAS screen showed nine hits against RAD51, which was the key member of the high-fidelity homologous recombination (HR) pathway, but only one hit against RAD52, which was part of the low-fidelity non-homologous end-joining pathway [42]. It was previously shown that the inhibition of the high-fidelity HR leads to the activation of the low-fidelity default microhomology end-joining repair pathway [68].

Thus, these many results clearly impacted and disrupted all the major functions of the chromosomes and likely provided a powerful epigenomic underpinning for the epidemiologically observed carcinogenic and teratogenic pathophysiology. Moreover, the DNA breakage was shown to be a prominent feature of the cannabis exposure of oocytes, sperm, lymphocytes and many other cells, and these finding imply that these lesions were preferentially repaired low-fidelity rather than high-fidelity pathways due to the epigenomic dysregulatory mechanisms.

### 3.3. Brain Development and Brain Aging

The Schrott EWAS study [42] revealed a widespread disruption to the receptor-based signaling, including 132 of the ionotropic AMPA receptors (GRIA), the main workhorse excitatory receptor of the CNS, 165 hits on the kainate glutamate receptor (GRIK), 26 hits on the NMDA glutamate receptor (GRIN) that mediates neuroplasticity and long-term potentiation, 11 hits on the delta glutamate receptor (GRID), 122 hits on the glutamate metabotropic receptor (GRM), 125 hits on the inhibitory GABA A receptor (GABRA), 22 hits on the GABA B receptor (GABRB), 85 hits on the “feel good” serotonin receptor (HTR), 17 hits on the dopamine receptors, five hits on each of the μ- and δ-opioid receptors and seven hits on the oxytocin “feel great” receptor.

There were ten hits each on neurexin and neuroligin, which are a ligand–receptor pair that mediate the receptor formation and scaffolding. There were eight hits on discs large homolog-associated protein 2 (DLGAP2), which is a protein known to be involved in synaptic scaffolding and the previously implicated ion autism development [102]. Similarly, there were 14 hits on the Down syndrome cell adhesion molecule (DSCAM), which is involved in axonal and dendritic pathfinding, self-avoidance and olfaction.

Recent studies have shown that the massive overgrowth of the human cerebral cortex relative to other species is controlled by signaling between the Slit-Robo ligand–receptor pair [199,200,201]. This was shown to be inhibited by cannabis [202,203,204]. There were 351 hits for the Slit signaling in the Schrott dataset and 40 hits for Robo. Moreover, there were eight hits for a key activating enzyme in this pathway—the Slit-Robo Rho GTPase activating protein (SRGAP2). These findings imply the impeded brain and neocortical outgrowth.

Another key study found that the very high gradient of retinoic acid at the frontal pole of the forebrain was responsible for driving the frontal lobe outgrowth [205]. The gradient was maintained using a retinoic acid synthesizing enzyme—alcohol dehydrogenase 1 (ALDH1) —at the frontal pole, transduced by the retinoic acid receptors RXRG and RARB, and was dissipated using the metabolic enzymes of the CYP26B1 group which had a high concentration at the posterior of the frontal lobe and the premotor cortex. There were 13 hits in the Schrott EWAS dataset for ALDH1, ten hits for RXRG and RARB and ten hits for the CYP2 series cytochrome metabolizing enzymes. 

These data showed that the cannabinoid stimulated epigenomic pathways disrupted the synaptic processing across a broad range of receptor subtypes, synaptic scaffolding using several routes, and neural progenitor and forebrain outgrowth by inhibiting several of the main pathways responsible for this key proliferation action. Such findings indicated that mental illness and congenital neurological conditions, including autistic spectrum disorders and developmental disorders such as microcephaly and anencephaly, were more likely, as observed in an increasing number of large epidemiological studies on community cannabis exposure [28,29,40,45]. Since these disorders were also characterized by impaired brain development, they may be seen as broadly degenerative in nature and, thus, consistent with an advanced broadly defined aging profile. 

This was, in turn, systemically important as brain aging has been well demonstrated to drive whole organism aging [164,165,166,206]. Indeed, accelerated systemic aging accompanies many syndromes where brain aging features prominently, including progeria and Down syndrome [207,208,209,210,211].

### 3.4. Vascular Aging

The issue of vascular aging has broader implications than simply the cardiovascular system since it has been aphoristically said that “you are as old as your arteries” [164,165,212,213]. This is true not only because most people succumb to macrovascular cardiovascular disorders [214] but because most stem cell niches contain a microvascular compartment which is key to stem cell function generally.

Cannabis exposure has been shown to advance human cardiovascular age in an ecological longitudinal study [33].

The key genes in arterial development are sonic hedgehog (shh), the vascular endothelial growth factor (VEGF) and notch and ephrinB2 signaling [215].

Importantly, when investigating the EWAS-identified epigenetic methylation changes to human sperm, sonic hedgehog signaling was shown to be disrupted by nine hits on both the patched co-receptor and elsewhere, in addition to 185 hits on the key Gli3 transcription factor which signaled to the nuclear genome [42]. Notch, VEGF and ephrinB2 were disrupted at 18, five and one hits, respectively [42].

The point of these findings was not only to identify that cardiovascular development can be disrupted in these ways but that the induced arterial aging can also induce the system-wide whole organism aging processes through an impairment of the stem cell quiescence/multiplication balance both directly and indirectly.

### 3.5. Epigenomic Disruptions by Organ System

The Schrott EWAS contained 73 hits for central nervous system dysfunctions, including the brain, neurological, synaptic, cerebral, neuronal and eye derangements.

At total of 29 hits were noted for cardiovascular disorders, including the heart, atria, ventricles, atrioventricular valves and vessels.

Additionally, 22 hits were noted for orofacial genetic lesions, including the head, sensory organs, palate, nose, anterior eye and ear derangements.

Six hits were identified for limb development directly. Further exploration of a more exhaustive list of the limb morphogens revealed 130 hits for most of the key morphogens involved in limb and digit development, including the fibroblast growth factors (FGFs), retinoid signaling, Wnt signaling, bone morphogenetic pathway signaling and five genes from the sonic hedgehog (shh) pathway, namely MEGF8 (multiple EGF-like domains 8), TMEM107 (transmembrane protein 107), Gli3 (GLI gamily zinc finger 3), CHD7 (chromodomain helicase DNA-binding protein 7) and the patched receptor cofactor. Indeed, 185 hits for the key shh transcription factor Gli3 were found in the Schrott EWAS.

There were 37 hits for development of the gastrointestinal tract, including references to the esophagus, large intestine, liver and pancreas. This epigenomic pattern was noted to be consistent with the pattern of the anomalies observed in the population cannabis exposure data from both the US and Europe [28,36,40,51].

There were 23 hits observed for the urinary system, including the kidneys. When a more detailed exploration of gene regulation guided by a recent developmental renal cell map was used as a guide for data mining [216], a total of 51 hits were identified for renal development. In addition 18, 27 and 18 hits were identified for the key renal tract morphogens—notch, sonic hedgehog and transforming growth factor β, respectively.

A total of 15 hits were identified for the body wall and embryo.

Additionally, 60 hits were noted for the general otherwise unclassified disorders, including embryonic growth, DNA, mitochondria, microtubules, body trunk, body axis, ovarian reserve, breast disorders, granulocytes, myogenesis, vertebral growth and bone development.

Hence, an abundance of epigenomic evidence exists to explain the broad spectrum and high severity of the teratological patterns observed in the many jurisdictions described. These findings are described in further quantitative detail elsewhere [217].

### 3.6. Cancer Hits in the Schrott EWAS

The Schrott supplementary file lists 487 hits for “cancer”, 112 hits for “tumor”, 126 hits for “carcinoma”, 36 hits for “neoplasm”, 32 hits for “leukemia” and 17 hits for “lymphoma”. This totals 810 hits for cancer and its synonyms, making this one of the standout and major findings of this EWAS report.

The report specifically mentioned many leukemias, lymphomas, myeloma and tumors of the breast, ovary colorectum, thyroid, liver, brain, pancreas, melanoma, stomach, esophagus and upper aerodigestive tract.

As noted, all of these cancers have been described in association with cannabis exposure in historical [22,23,24,25,26,30,41,51,218,219,220,221,222,223,224,225,226,227,228] and recent reports [22,23,24,26,30,34,41,51,83,229]. Further quantitative details in the description of this material is provided elsewhere [229].

By listing over 30 cancers by name and the genes whose epigenomic modulation was linked with them, these results provided a powerful pan-cancer mechanistic contributory explanation for the patterns of cancer epidemiologically observed in human populations.

It is also worth noting that many of the more recent epidemiological reports proceeded beyond the methodologies commonly adopted in observation cohort studies [22,23,24,26,30,34,41,51,83,229,230]. By applying the formal techniques of causal inference including inverse probability weighting and E-values to quantitatively exclude extraneous unmeasured confounding these investigators have constructed a pseudo-randomized analytical framework and, therefore, reported the causal relationships in preference to the more commonly noted ecological associations [22,23,24,26,30,34,41,51,83,229,230].

### 3.7. Aging Implications of the Schrott EWAS Dataset

A concise overview and introduction to aging was provided in the preceding sections. Since DNA methylation was shown to be a key determinant of the progressive decline of the function which characterizes the aging process, and since cannabis dependence and withdrawal was shown to widely disrupt both DNA methylation and demethylation and the histone code with which it is coordinated, the disruption of the aging process itself is not unexpected. As noted above, this was confirmed in somatic tissues experimentally and found to be of high magnitude at 30% at 30 years of age [54].

It was also shown that cannabinoids can reduce the telomerase activity in a rat hepatocarcinogenesis model [121]. The Schrott EWAS dataset showed that the telomerase activity was epigenetically reduced with a significance of *p* = 2.82 × 10^−6^ and a multiplicity-corrected *p*-value of 0.01258 [42]. Indeed, since cannabis dependence inhibits TET1 (*p* = 1.18 × 10^−5^, multiplicity-corrected *p*-value = 0.02278) and this is the main counterbalancing force to the promoter hypermethylation of aging, it is easy to understand how the accelerated aging process is not only established initially, but how it might become a positive feed-forward process with time as age-related epigenomic changes are predisposed to further pro-aging epigenomic processes.

Two of the key tissues with which we were concerned in the present context were the male and female gametes. It was understood that none of the presently available epigenetic clocks were suitable for the application to measure the relatively very hypomethylated ages of the gametes. However it does stand to reason that it may be possible to develop such an algorithmic clock mathematically. What the negative ages might mean, as they may relate to ages prior to birth, has yet to be determined biologically. Hence, it was not possible to measure gametal age directly or epigenomically at the time of writing.

However, it was emphasized that the well-described presence of the characteristic aging nuclear changes on the sperm of the DNA chromosomal breaks and translocations [115] and oocyte nuclear blebs and bridges [114] provided strong genetic evidence of the changes of accelerated aging. The likelihood then that both gametes and the fertilized zygote are “old prior to conception” clearly has far reaching public health and multigenerational implications in terms of the prenatal origin of many disorders in later life [171,231].

### 3.8. Strengths and Limitations

There are various strengths and limitations to the present conceptualization. The strengths include the remarkable consistency across the many epidemiological studies, which clearly demonstrates the genotoxic harms of cannabis exposure in several different international jurisdictions, in relation to both the congenital anomalies [28,36,37,40,45,46,49,51,71,73,74,75,76,77,78,79,80,81,82] and cancer [22,23,24,25,26,30,41,232], and, indeed, now also in aging [53,54]. Similar results in many different studies are clearly mutually supportive and strengthen the overall quality of the body of evidence. Similarly, there is a striking concordance between the many epigenomic studies of gestational cannabis exposure in relation to global DNA hypomethylation and the disruption of DNA methylation levels at key promoter and enhancer sites, which control the regulation of many critical genes [42,101,102,103,105,106,107,108,233,234]. 

The major limitation of the present work is its preliminary nature in that we present an introductory conceptual framework which needs to be filled out and completed by numerous further laboratory studies. The purpose of the present paper is merely to draw attention to this remarkable concordance of cross-disciplinary results and indicate to researchers in the basic sciences that the field is ripe for detailed exploration in many studies with far-ranging consequences. 

### 3.9. Future Directions

In the same way that Harvard researchers were recently able to advance cellular and organismal age forwards and backwards by the induction of just a few DNA breaks and then demonstrate their phenotypical rescue with the OSK Yamanaka stem cell factors [235] so too models of cannabis exposure lend themselves to similar exploration by experimental induction of aging in cells and model organisms and then their rescue either with a subset of the Yamanaka factors [184,185,186,187,188,235] or chemical cocktails [236], which are similarly directed. Thus, cannabinoid research could intersect powerfully with aging research in general at a time when the whole field is making important advances.

Moreover, the 810 differentially methylated genes implicated with cancer by the work of the Murphy lab [42] have not yet been explored. In much the same way that potentially important breakthroughs may occur by exploring and developing cannabis-based models of aging, they could also be developed in cancer. While the present paper serves to sketch a general outline based on the now considerable body of evidence from DNA methylation work, there is much of the story which urgently needs to be investigated. Due to its obvious public health importance from the widespread and now intergenerational cannabinoid exposure, which is occurring in many places, it is appropriate that cannabinoid oncogenesis serves as an important model for cancer research.

The important questions that need to be considered include the following.

- How is the landscape of the post-translational histone modifications changed by cannabis exposure, particularly in relation to the key modifications, such as histone acetylation, which reliably mark active genes?
- How are the critically important issues of the modulation of super-enhancers and super-anchors affected by cannabinoid exposure?
- Can tissue de-differentiation be demonstrated experimentally from cannabinoids and, if so, which tissues are the most susceptible? What are their time course? What are their dose-response effect? In particular, in the ovaries, testes, brain, liver, heart, respiratory tract and immunocytes. Is this de-differentiation premalignant? Do cannabinoids induce premalignant field changes and cancerization from adverse genomic (DNA breaks), epigenomic or metabolomic effects or some interaction between all of these and more?
- Oocytes are particularly genomically fragile and non-renewable cells and have a very long life of many decades. Their genomic, metabolic and epigenomic vulnerability to cannabinoids needs to be characterized in detail. What are the effects of cannabinoids on oogenic stem cells both prenatally and postnatally?
- Since cannabinoids affect the mitochondria and cell metabolism adversely and this is closely related to maintenance of both the genome and epigenome, how does this compare to the Warburg effect observed in stem cells and cancer cells [237]? How is it similar? How is it different?
- Such studies would provide an invaluable window of insight into the way the metabolome and epigenome are coordinated and bidirectionally co-regulate each other.
- In particular, the effect of lactylation as a key post-translational modification of the key metabolic enzymes and histones needs to be quantified, as this has also been shown to be a critical post-translational modification for stem cells, cancer cells and cancer stem cells with potentially far-reaching and cross-disciplinary applications in aging medicine and cancer biology [238,239].
- As described, cannabis widely disrupts many of the key enzymes of the epigenetic machinery itself. What are the implications of this?
- As discussed, cannabinoids disrupt key elements of the mitotic spindle, microtubule physiology, spindle pole formation and kinetochore and centrosome formation. These are very far-reaching findings as chromosomal mis-segregation has implications in many fields, including male and female fertility optimization, sperm and egg storage, the preservation and fertility of medicine, the biology of aging, cancerogenesis, congenital anomalies in early pediatrics and neurodevelopmental alterations in later pediatrics. This field has been largely ignored but is very much in need of detailed exploration and explication at the cellular and molecular levels from the point of view of the impacts of cannabinoids.
- As described by epigenomically disrupting both CTCF and the major components of the cohesion complex, it seems inevitable that cannabis must disrupt the basic machinery of chromatin looping and gene expression itself. What are the implications of this potentially very far-reaching derangement?
- What is it about testicular germ cells that makes them particularly susceptible to cannabinoid oncogenesis? Is it the DNA hypomethylation of germ cells to start?
- Techniques are emerging to allow the characterization of the non-protein coding, regulatory and repeat sequences in the genome. In what way is this normal physiology perturbed by cannabinoid exposure?
- DNA hypomethylation of the gene bodies and gene deserts is likely to result in the mobilization of transposable elements in the genome, which precipitates genomic instability, cytoplasmic and extracellular immune activation (via cGAS-–STING and downstream IL1β, IL6 and interferons) and, thus, aging and oncogenesis in both the immune and genomic pathways. This field needs to be explored and further developed.
- The advancement of human epigenomic age from cannabis exposure at a single age has been mentioned [54]. What is the time course of this across the lifespan? How does it progress? Dose it rise as a linear function of age or as a polynomial or exponential function of age as suggested by the biophysical clinical studies [33]? What are its dose-response characteristics?
- Many different cannabinoids need to be profiled in vitro to characterize their multichannel epigenomic effects (DNA methylation, many histone methylation and acetylation modifications, super-enhancers) in the modern era to define the breadth of their epigenotoxicity as a possible or likely class effect.
- The well-documented exponentiation of the cannabinoid dose-response effects in many cellular and metabolic assays needs to be formally explored in the modern epigenomic context and its public health significance needs to be carefully considered.

These and a host of similar questions are now present for careful review and exploration by modern laboratory techniques with almost certainly far-reaching implications for both the basic sciences and clinical medicine.

## 4. Conclusions

The above overview of several large datasets is notable for many reasons. There is a surprising uniformity in the findings from the three domains of cannabinoid-related teratogenesis, carcinogenesis and the acceleration of aging processes, which together imply a clinically significant if widely underrated cannabinoid genotoxicity. Moreover, the severity of the findings in all three domains is considerably greater than is commonly supposed.

The other very striking feature is that the predictive power of the epigenomic profiles provided by the recent epigenome wide studies, particularly that of Schrott and colleagues [42], is the predictive power of the morbidity data arising from both the European and US datasets following increased cannabis exposure. The identified DNA methylation changes and associated biological mechanistic pathways explain these observed patterns of disorders.

The wide spectrum of cancers is specifically explained and the organ-specific nature of many congenital anomalies is explained. The pathophysiology of the wide spectrum of chromosomal disorders which occur as both malignant disorders and congenital anomalies is also elegantly explained by several mechanisms. The pattern of limb anomalies, including syndactyly and polydactyly, is explained by the widespread epigenomic disruption of most of the key limb morphogen gradients. Most particularly, the patterns of accelerated aging are explained by the epigenomic inhibition of DNA methylation, demethylation and telomerase, among others, and confirmed by the recent pattern of chronic illness studies and epigenomic clock investigations.

Most concerning, the very real prospects that both sperm and oocytes are epigenomically aged prior to conception carry severe and dire prospects for public health and multigenerational epigenomic transmission and beg a formal assessment using gamete-appropriate epigenomic clocks whose development must be considered a major research priority.

With the increasing interest in the pharmaceutical use of cannabis and cannabis derivatives for medical management, it is increasingly important that the balance between the therapeutic risks and benefits for each potential cannabis/cannabinoid application is thoroughly understood. The mandate of regulatory global authorities is to not succumb to strong commercial and popular interest in the promotion of cannabis/cannabinoids until the risk–benefit appraisal can be accurately undertaken for each potential cannabis application, considering, among other items, the potency, severity of disease, stage of human development and duration of use.

## Data Availability

Not applicable.

## Abbreviations

Abbreviation	Meaning
ALDH1	Alcohol dehydrogenase 1
ALL	Acute lymphoid leukemia
AML	Acute myeloid leukemia
AMPA	α-amino-3-hydroxy-5-methyl-4-isoxazolepropionic acid
CENPA	Centrosomal protein A
CENPN	Centrosomal protein N
CHD7	Chromodomain helicase DNA-binding protein 7
CYP	Cytochrome
DLGAP2	Discs large homolog-associated protein 2
DSCAM	Down syndrome cell adhesion molecule
EMA	European Medicines Agency
EWAS	Epigenome-wide association study
FDA	Food and Drug Administration
FGF	Fibroblast growth factor
GABA	γ-aminobutyric acid
GABRA	GABA A receptor
GABRB	GABA B receptor
Gli3	GLI family zinc finger 3
GRIA	Glutamate ionotropic receptor AMPA-type subunit
GRID	Glutamate ionotropic receptor Delta-type subunit
GRIK	Glutamate ionotropic receptor Kainate-type subunit
GRIN	Glutamate ionotropic receptor NMDA-type subunit
GRM	Glutamate metabotropic receptor
HTR	Serotonin receptor
Klf4	Kruppel-like factor 4
MEGF8	Multiple EGF-like domains 8
MHCPRA	Medicines and Health Care Products Regulatory Agency
Myc	Myc proto-oncogene, bHLH transcription factor
NMDA	N-methyl-D-aspartate
NUMA	Nuclear-mitotic apparatus protein
Oct3/4	POU5F1, POU class 5 homeobox 1
RAD51	RAD51 recombinase
RAD52	RAD52 homologue, DNA repair protein
RARB	Retinoic acid receptor beta
RSC	Remodelers of the structure of Chromatin
RXRG	Retinoid X receptor gamma
shh	Sonic hedgehog
SMARCA	SWI/SNF-related, matrix-associated, actin-dependent regulator of chromatin, subfamily A
Sox2	SRY-box transcription factor 2
SRGAP2	Slit-Robo Rho GTPase-activating protein 2
TET1	Ten-eleven translocase
THC	Δ9-tetrahydrocannabinol
TMEM107	Transmembrane protein 107
UHRF	Ubiquitin-like with PHD and ring finger domains
VEGF	Vascular endothelial growth factor
Wnt	Wnt family member
Δ8THC	Δ8-tetrahydrocannabinol
Δ9THC	Δ9-tetrahydrocannabinol
